# Fine-grained identification of greenhouse crop leaf diseases based on reconstruction-generation network

**DOI:** 10.1371/journal.pone.0343228

**Published:** 2026-03-09

**Authors:** Yang Wu, Jie Liu

**Affiliations:** 1 School of Intelligent Equipment Engineering, Wuxi Taihu University, Wuxi, China; 2 Wuxi Key Laboratory "Intelligent Robot and Special Equipment Technology Key Laboratory", Wuxi Taihu University, Wuxi, China; 3 "Complex Discrete Manufacturing Intelligent Equipment Technology" Key (Construction) Laboratory of Universities in Jiangsu Province, Wuxi Taihu University, Wuxi, China; PLOS, UNITED KINGDOM OF GREAT BRITAIN AND NORTHERN IRELAND

## Abstract

There are few data labels in the agricultural field, and accurate annotation of existing data requires professional knowledge and is time-consuming and laborious, especially the images collected in the actual greenhouse scene, which lack accurate annotation by professionals. Fine-grained refers to the highly detailed division or analysis of data or tasks, with a particular emphasis on capturing micro-level differences. In order to improve the accuracy of greenhouse crop disease identification, the crop disease identification problem is regarded as a fine-grained classification problem, and the attention mechanism is introduced into the classification network. The VAE enhancement strategy is introduced into the disease identification network model to improve the accuracy when the annotation is insufficient. Aiming at the problem that the actual environmental background of greenhouse is complex, there are many disturbances, the disease spot area is small, and the difference between leaf disease and wilt and soil is not obvious, a fine-grained identification model of leaf disease based on reconstruction-generation is further proposed. The attention mechanism was used to increase the recognition ability. During training, the VAE strategy was first used to make full use of a large number of labeled and unlabeled data to realize unsupervised learning, and then the labeled data was used for supervised disease identification, and the Reconstruction-Generation Network(RGN) was used to force the classification network to pay more attention to discriminative regions to find differences. Reconstruction-generation belongs to self-supervised learning, which uses the unsupervised information in the data to construct supervised signals, and can generate useful feature representations by learning the structure and pattern in the data. Experimental results show that the classification recognition accuracy of the proposed fine-grained leaf disease identification model based on reconstruction-generation adopts the attention mechanism reached 98.03%. The proposed method is applied to the detection model, the correct recognition rate of diseased leaves was 95.07%, and the correct recognition rate of healthy leaves was 98.46%, which can realize the end-to-end detection and identification of diseased leaves and meet the practical requirements.

## 1. Introduction

In recent years, the research on crop disease and insect pests recognition using deep convolutional networks has received extensive attention. However, in actual agricultural production, especially in complex scenes such as greenhouses, the recognition rate is not satisfactory. Greenhouses are different from fields, which generally have insect-proof nets, which are difficult for insects to enter, and pests rarely occur. However, due to the characteristics of high temperature and high humidity, once the diseases occur, they will spread quickly. Because of the complex light environment caused by sunlight changes and mutual occlusion of buildings or plants in greenhouses, the color of crops and disease spots is close to the surrounding background, and the image quality obtained by low-cost cameras is poor, it is difficult to identify greenhouse crop diseases.

Most of the current research using deep learning technology is based on public datasets such as PlantVillage [1], and other available datasets are few, which is difficult to meet the needs of practical applications. Mohanty et al. [2] used a public dataset from PlantVillage, images of diseased and healthy plant leaves taken under controlled conditions, to train a deep convolutional neural network to identify 26 diseases in 14 different crops. Based on transfer learning, Wang et al. [3] proposed a crop disease classification model TL-SE-ResNeXt-101 by improving the SE-ResNeXt-101 model, which was used for disease recognition of crop types in the reconstructed AI Challenger 2018 dataset. In addition, some scholars have used deep learning technology to study the disease identification in greenhouse scenarios. For example, Gutierrez et al. [4] created a dataset containing a large number of images of infected tomato plants to generate and evaluate machine learning and deep learning models to select the best method based on pest detection and identification accuracy. Zhang et al. [5] proposed a tomato disease diagnosis method based on residual learning of Multi-ResNet34 multimodal fusion learning, which integrates multi-source data (tomato disease image data and environmental parameters), introduces transfer learning, speeds up training, reduces data dependence, and prevents overfitting caused by a small amount of sample data. Fuentes et al. [6] proposed a paradigm called target class control to help improve the generalization ability of the model, and a strategy to improve the accuracy of the model by dealing with complex changes in the new greenhouse environment. Zhang et al. [7] obtained a cucumber leaf disease dataset in a natural complex greenhouse background and constructed a classification model using the currently advanced method EfficientNet-B4. Fatima et al. [8] designed an intelligent greenhouse system based on the Internet of Things (IOT), which realizes the combination of monitoring, alerting, cloud storage, automation and disease prediction, and immediately remedied when abnormal conditions occurred, and an effective deep learning model was used for disease recognition of leaf images. Liu et al. [9] constructed a cucumber disease dataset with complex background, and used two methods to reduce the computational energy consumption of the model and change the feature extraction calculation method, so as to extend the working time of the greenhouse edge equipment where the disease model was deployed. Bai et al. [10] comprehensively summarized the current status of DL in disease vector identification, covering data collection, data preprocessing, model construction, evaluation methods, as well as applications ranging from species classification to object detection and habitat identification. Hu et al. [11] proposed DepMulti-Net, which is a novel model for rice pest and disease recognition. It aims to overcome the challenges of complex background interference, difficulty in extracting disease features, and large model parameter quantity in rice leaf disease recognition. The results show that the DepMulti-Net model has only 13.5 million parameters, and the average accuracy rate reaches 98.56% when identifying four types of rice diseases. It provides an efficient and lightweight solution for crop disease recognition.

Compared with traditional methods, deep learning methods do not need to manually design features, but automatically learn features from large data sets, which provides another idea for crop disease recognition. Training deep learning models requires a large number of labeled sample data to ensure the generalization performance of the model to new data, and it has a high image identification rate when the sample is sufficient. The method based on deep learning can extract the global feature information of the image, but in complex scenes such as greenhouses, it is difficult to accurately label the collected images, so the number of labeled samples is small. The supervised deep learning method for crop disease identification still has shortcomings, and most supervised deep learning methods are not satisfactory in terms of identification accuracy and speed. Most of the current supervised deep learning methods are not satisfactory in terms of recognition accuracy and speed. At present, many existing convolutional neural network (CNN) methods have complex structures and large parameters, which cannot achieve satisfactory results in practical applications, and are difficult to be applied to similar agricultural applications with insufficient samples. Compared with the amount of data in public datasets, the amount of actual data collected is much less. Therefore, much of the research in this paper is based on the public datasets, and related research is done according to the actual datasets to apply to the greenhouse scene. In summary, for the specific scene of greenhouse, studying the image recognition technology of crop diseases, improving the accuracy of disease recognition, early diagnosis and treatment, has important theoretical value and practical significance, and has broad application prospects. Fine-grained refers to the highly detailed division or analysis of data or tasks, with a particular emphasis on capturing micro-level differences. This concept is widely applied in fields such as computer vision and natural language processing. Fine-grained image classification, also known as sub-category recognition, differs from the general image task in that the granularity of the image categories is much finer. Its aim is to make more detailed sub-classifications within broad coarse-grained categories. However, due to the subtle inter-class differences and significant intra-class variations among sub-categories, fine-grained image classification is more challenging than ordinary image classification tasks.

The key contributions of this study are summarized as follows:

(1) The problem of crop disease identification is regarded as a fine classification issue, and the attention mechanism and VAE enhancement strategy are introduced into the classification network to improve accuracy in the case of insufficient labeling.
(2) A fine recognition model for greenhouse crop leaf diseases based on reconstruction-generation is proposed. The RGN is used to force the classification network to pay more attention to the distinctive regions, in order to discover the differences.
(3) The model does not participate in the calculation during the inference stage, which will not introduce additional computational overhead and storage consumption in the actual model deployment and production environment.
(4) When applied to the detection model, the proposed method can improve the accuracy of crop disease detection and can effectively be extended to similar disease recognition applications with smaller data volumes.

Different from previous studies, our method effectively enhances feature representation through the attention mechanism, improves accuracy by using unsupervised data through the VAE enhancement strategy, and guides the network to focus on local regions through the reorganized generation model. The results show good performance.

The main content of this article is divided into the following four section: Section 1 is “Introduction,” which first introduces the research background and significance of this paper, the current research status, presents the contributions of this paper, and lists the organizational structure of the paper. Section 2 is “Materials and methods,” which introduces the data set, preprocessing, and focuses on the proposed disease identification model. Section 3 is “Results and discussion,” which presents the experimental setup, implementation methods, and related research data of this paper. Section 4 is “Conclusions,” which summarizes this paper.

## 2. Materials and methods

### 2.1. Dataset

Deep learning usually requires a large number of training examples to cover the entire sample space. The number of samples needed for deep learning depends on factors such as task complexity, data type, and data distribution. For some simple tasks such as digit recognition, only a few thousand samples may be needed to achieve high accuracy. But for some complex tasks, such as image classification and natural language processing, tens to hundreds of thousands or even more samples are usually needed to achieve good performance. In this chapter, two datasets are used. One is the public dataset PlantVillage [1], which consists of images and corresponding disease categories, and is used as the training of the classification network model and the backbone network pre-training of the detection network model. The other is a tomato leaf dataset TJ-Tomato collected from real scenes. These images are labeled with disease labels and disease leaf regions, which are used for training and validation of the detection model.

**Fig 1:**
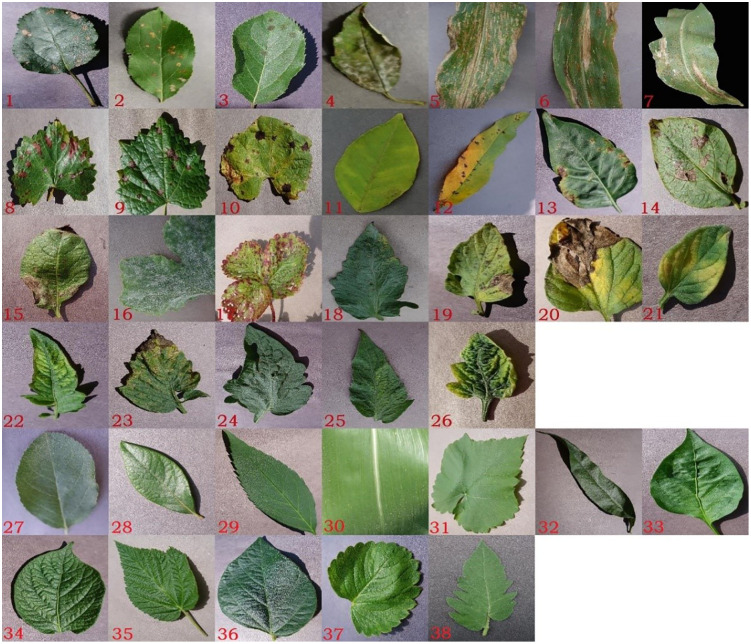
26 types of diseased leaves and 10 types of healthy leaves in PlantVillage [1].

PlantVillage is an internet plant disease image library initiated by David, an epidemiologist at the University of Pennsylvania, in order to solve the problem of plant disease diagnosis by using machine learning technology. The dataset collects 54303 visible leaf images of 38 categories of labels of 14 plants, including 12 categories of healthy images and 26 categories of leaf images with diseases, as the training data set for crop disease classification required for the experiment. Fig 1 shows examples of 26 categories of diseased leaves and 10 categories of healthy leaves in this dataset. No. 1–3 are black rot, rust and scab leaves of apple, 4 are cherry powdery mildew leaves, 5–7 are corn gray leaf spot, leaf blight and rust leaves, 8–10 are grape black measles, black rot and leaf blight, 11 are orange huanglongbing leaves, No. 12 is peach bacterial spot leaves, 13 is pepper bacterial spot, 14–15 are potato leaves with early blight and late blight, 16 are squash powdery mildew leaves, 17 are strawberry leaf scorch leaves, 18–26 are tomato leaves with bacterial spot, early blight, late blight, leaf mold, mosaic virus, septoria leaf spot, target spot, two spotted spider mite and yellow leaf curl virus diseases respectively, 27–38 are all healthy leaves, they are apple, blueberry, cherry, corn, grape, peach, pepper, potato, raspberry, soybean, strawberry and tomato respectively.

**Fig 2:**
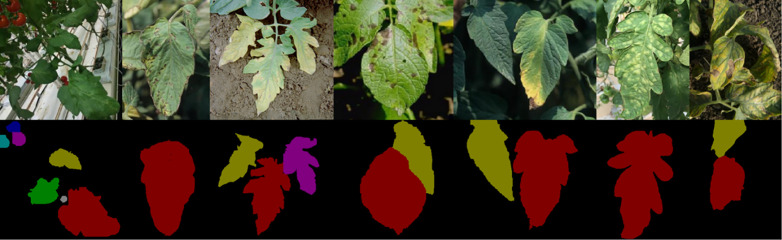
Pictures of a tomato leaf from a real scene.

The TJ-Tomato dataset is a data set containing healthy and diseased tomato leaves taken in a glass greenhouse, which is located in Chongming Experimental Base of China National Facility Agricultural Engineering and Technology Research Center. The pan-tilt-zoom (PTZ) camera is used to move according to the preset path and realize fixed-point image acquisition. The dataset was taken by A Hikvision DS-2DC7520IW-1 (5 million pixels) PTZ camera to monitor the growth of tomato, and the 2592 × 1944 resolution pictures were obtained. The pictures were collected from 9 am to 5 pm. Due to the strict management in the glass greenhouse, the disease occurred less by using matrix culture. Due to the strict management in the glass greenhouse, the disease can be detected and eliminated in time, so that the actual collection of disease data is less. After screening, the data set contains 463 healthy leaf pictures and 186 pictures with disease leaves. As shown in the first row of Fig 2, in the figure, (a) is the picture of healthy tomato leaves, (b)-(g) are the pictures of leaves with diseases, which are tomato bacterial spot (TBS), tomato early blight (TEB), TEB, tomato late blight (TLB), tomato leaf mold (TLM) and tomato septoria leaf spot (TSLS).

### 2.2. Preprocessing

In order to prevent overfitting, enhance the robustness and reliability of the model, and improve the versatility of the classification model, taking tomato disease as an example, the distribution of the sample number of each category in the original data set is unbalanced. Some categories have too many samples, and some have too few samples. In order to speed up the calculation and balance the distribution of various samples, 38 types of healthy and diseased leaves in the PlantVillage dataset were normalized to 128 × 128 × 3, and each type was randomly divided into training set and validation set according to the ratio of 6:2:2. The test set was used to train the model, select hyperparameters, and test accuracy. The number of samples in the training set was divided into five groups according to the proportion (the proportion of labeled training set and unlabeled training set in each group was different), and the model was trained under different conditions and the performance of the model was evaluated. The proportion of labeled training set to unlabeled training set in the five groups was 10%−90%, 30%−70%, 50%−50%, 70%−30%, and 90%−10%, respectively, to verify the effect of the recognition method under different circumstances. The number of samples in the five groups is shown in Table 1.

**Table 1:** Division of datasets.

Serial No.	Validation set-20%	Testing set-20%	Training set-60%(32581)			
	Quantity	Quantity	Labeled		Unlabeled	
			Proportions	Quantity	Proportion	Quantity
1	10861	10861	10%	3258	90%	29323
2	10861	10861	30%	9774	70%	22807
3	10861	10861	50%	16291	50%	16290
4	10861	10861	70%	22807	30%	9774
5	10861	10861	90%	29323	10%	3258

Due to the limitation of the collection scene, the TJ-Tomato dataset consists of 463 images of healthy leaves and 186 images of diseased leaves. In the second row of Fig 2, the labels used to train the segmentation network after instance segmentation are used, and different colors are used to distinguish different target instances. The images with only disease leaves in TJ-Tomato dataset were expanded by horizontal mirror and 180 degree rotation, and 558 disease images were obtained after expansion. The images of healthy leaves and diseased leaves were divided into the training set and the test set according to the ratio of 7:3, that is, 324 images of healthy leaves and 391 images of diseased leaves were selected as the training set to train the detection model, and the others were used as the test set to verify the effect of the method. The test set included 167 images of diseased leaves and 139 images of healthy leaves.

### 2.3. Fine-grained identification of greenhouse crop leaf diseases

#### 2.3.1. Overall structure.

**Fig 3:**
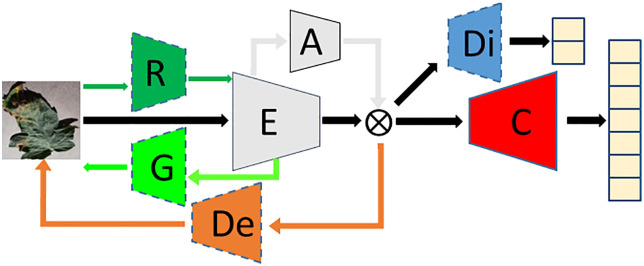
Structure of greenhouse crop leaf recognition model based on reconstruction-generation.

**Fig 4:**
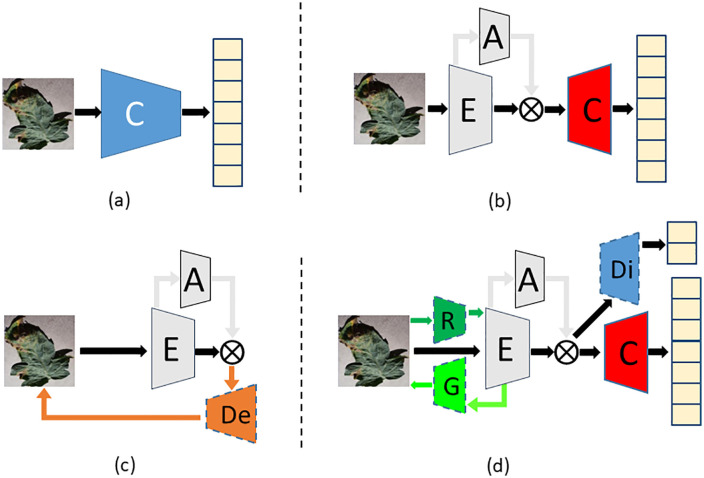
Schematic representation of the decomposed structure.

In order to improve the accuracy of leaf disease identification in greenhouse, a fine-grained identification model of greenhouse crop leaf diseases based on reconstruction-generation is proposed, as shown in **Fig 3**. Aiming at the problem that the disease spot area of greenhouse crop is small, and the difference between it and the dead leaves, soil and substrate is not obvious, and the disease recognition rate is not high, the crop disease recognition is regarded as a fine-grained classification problem, and the attention mechanism is introduced into the disease recognition model, as shown in **Fig 4(b)**. The attention mechanism is used to enhance the identification ability in the classification network, which has fewer parameters while improving the accuracy. Aiming at the problem that the images collected by the actual greenhouse scene lack accurate annotation by professionals, the VAE enhancement strategy (as shown in **Fig 4(c)**) is first adopted during training to improve the recognition accuracy when the annotation is insufficient. The enhancement strategy uses labeled and unlabeled samples to realize unsupervised learning, and then through the RGN, The classification network is forced to pay more attention to the discriminative region to find the difference, and the adversarial loss is applied in the discriminative network to distinguish between the generated image and the original image (as shown in **Fig 4(d)**).

**Fig 5:**
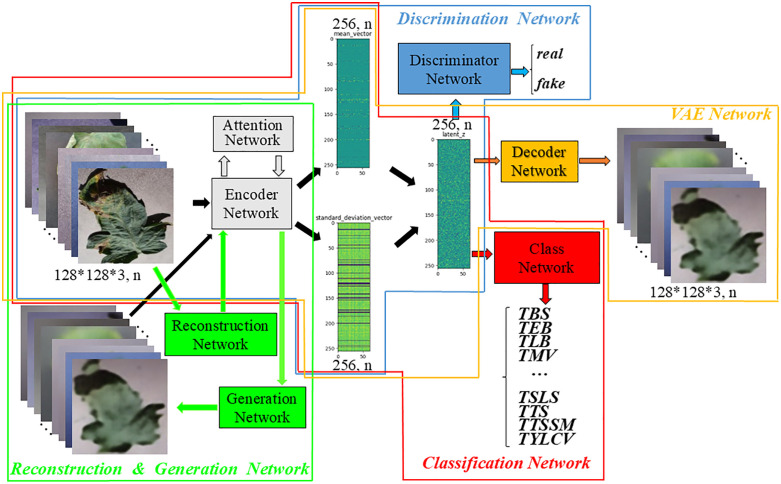
Greenhouse crop leaf recognition model based on reconstruction-generation.

Fig 3 shows the overall structure of the proposed fine-grained recognition model for greenhouse crop leaf diseases based on reconstruction-generation. On the basis of 4(b), decoder network “De”, reconstruction network “R”, generation network “G” and discrimination network “Di” are added, which are divided into three parts when used, namely VAE enhancement strategy (Fig 4(c)). When used, it is divided into three parts: VAE enhancement strategy (Fig 4(c), used only for training), RGN (Fig 4(d), used only for training), and identification network (Fig 4(b), used for inference). In Fig 4, (a) is the traditional classification model, and “C” represents the classification network. (b) is the disease recognition model using attention mechanism, which is the model finally used for inference. The above classification network is divided into two parts: encoder network (for feature extraction) and classification network, denoted by “E” and “C” respectively, and the attention network is added, denoted by “A”. (c) is the adopted VAE enhancement strategy, and all data are used to train the network structure shown in (c) during training, which can make full use of unlabeled samples to realize unsupervised learning and enhance network learning ability. (d) for the RGN model, the labeled data is used to train the disease identification model based on the RGN during training, forcing the classification network to pay more attention to the discriminative region to find the difference and improve the classification performance. After (d), the trained model was obtained. During inference, the “De”, “R”, “G” and “Di” networks were removed, and only the recognition network was retained, that is, the network model shown in (b) was used to identify the types of greenhouse crop leaf diseases. Fig 5 shows the greenhouse crop leaf identification model based on reconstruction-generation. The red box on the way is the classification model, the orange box is the VAE enhanced strategy model, and the green box is the RGN, including two parts: the RGN and the discrimination network.

#### 2.3.2. Identification model based on attention Mechanism.

Fig 4 (b) is the disease recognition model using attention mechanism, which is composed of three parts: encoder network “E”, attention network “A” and classification network “C”. The encoder network is used to extract the input image features, and two vectors of length 256 are obtained after the encoder network, and then the hidden vector z is obtained by reparameterization technique sampling. The hidden vector is not a simple reconstruction of the original data, but also introduces noise, which increases the generalization ability of the model. After that, the classification network determines the category. In the inference stage, the hidden vector z is directly replaced by the hidden vector μ instead of the reparameterization technique.

**Fig 6:**
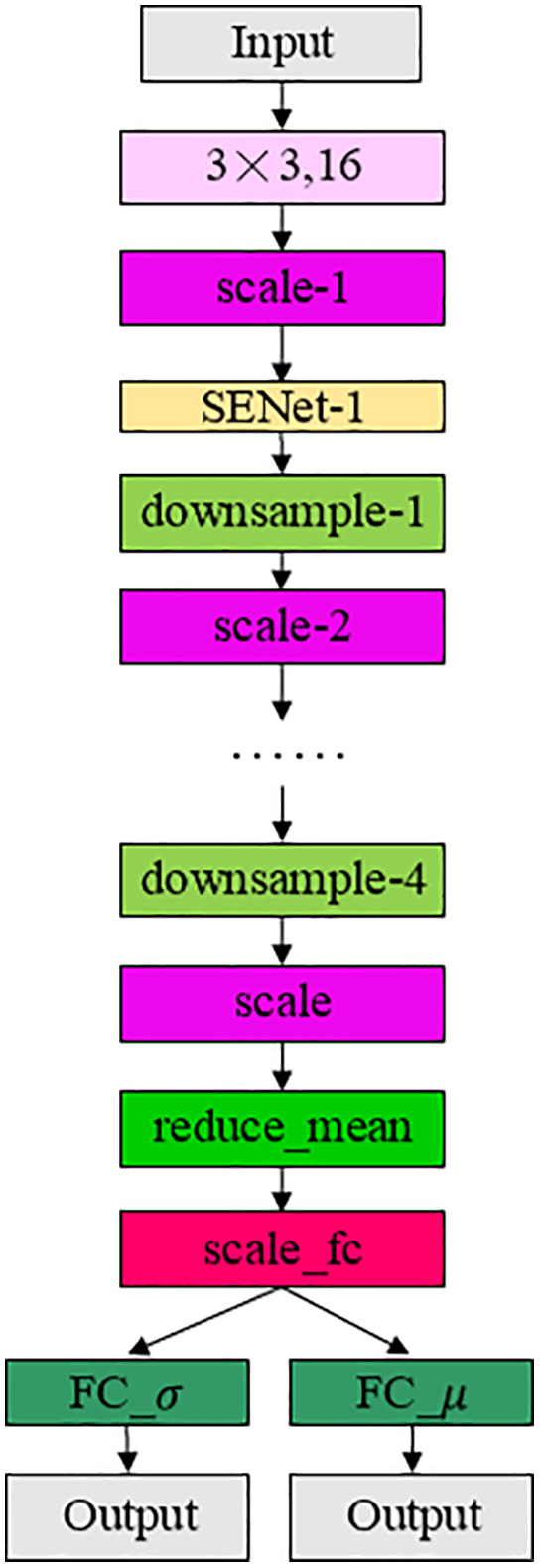
Encoder network with attention network.

The encoder network is shown in Fig 6. The encoder converts an input image X of size 128 × 128 × 3 into two vectors of size 256: mean and variance. The input and output sizes of each layer are shown in Table 2, “E” consists of a series of convolution layers. It consists of “conv”, 4 layers(consisting of repeated four layers of “scale”, “SENet” and “downsample”), as well as “scale”, “reduce_mean”, “scale_fc”, and two “FC”. Four layers is composed of three modules, “scale”, “SENet” and “downsample”, repeated four times in turn, “scale” is the improved ResNet module, which is used to extract features, “downsample” is used to reduce the size of each feature map and increase the number of channels, after each layer, the number of channels is doubled and the size is halved, “SENet” is the adopted attention mechanism network. The input of the model is a 128 × 128 × 3 image, after the conv layer, the size of the vector becomes 128 × 128 × 16, after 4 layers, the size becomes 8 × 8 × 256, “reduce_mean” is global pooling, and finally after the “FC” layer, two hidden vectors of size 256 are generated.

**Table 2:** Input and output dimensions of each layer of encoder and attention network.

Layer	Input	Output
conv(3 × 3, 16)	128 × 128 × 3	128 × 128 × 16
scale-1	128 × 128 × 16	128 × 128 × 16
SENet-1	128 × 128 × 16	128 × 128 × 16
downsample-1	128 × 128 × 16	64 × 64 × 32
scale-2	64 × 64 × 32	64 × 64 × 32
…	…	…
downsample-4	16 × 16 × 128	8 × 8 × 256
scale	8 × 8 × 256	8 × 8 × 256
reduce_mean	8 × 8 × 256	256
scale_fc	256	256
FC	256	256

**Fig 7:**
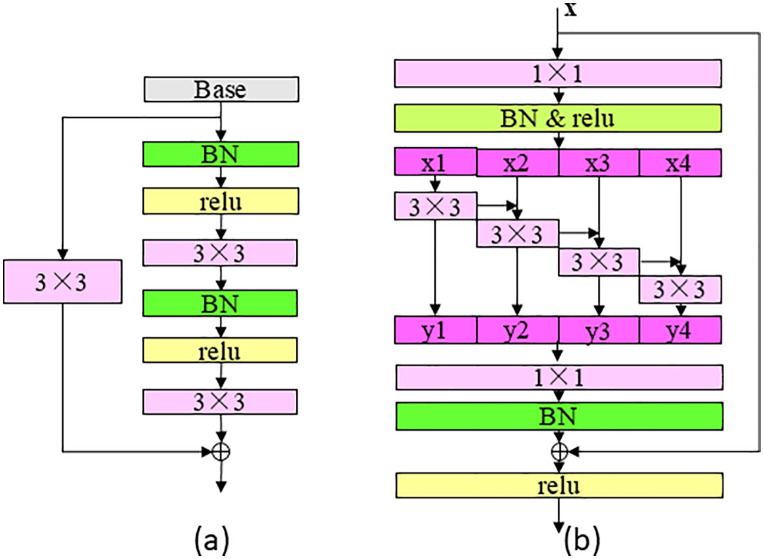
Structure of scale and grouped convolution 3 × 3 network.

**Fig 8:**
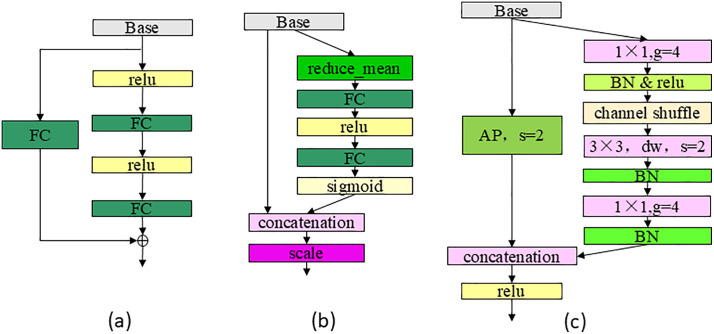
Structure of scale_fc, SENet and downsample.

“scale-1” to “scale-4” are all composed of scale layers, whose structure is shown in Fig 7(a) and is used to extract features, and the 3 × 3 is the grouped convolution structure shown in Fig 7(b), which is used to increase the network receptive field and network depth, and strengthen the feature extraction ability. The structure of “scale_fc” is shown in Fig 8(a) to better obtain global information. “SENet”, shown in Fig 8(b), is the attention mechanism adopted in this paper. The “downsample” module structure is shown in Fig 8(c), where “AP” stands for “average_pooling”, “s=2” stands for “stride=2”, that is, step size is 2, which is used to quickly reduce the size of the feature layer, “g=4” stands for grouped convolution, which means divided into 4 groups. channel shuffle refers to the channel rearrangement mechanism, and the specific operation is shown in reference [12]. “ShuffleNet” uses grouped convolution and channel rearrangement to achieve network acceleration. When grouping convolution, the number of channels in each convolution kernel operation is reduced, which can greatly reduce the amount of calculation. The classification network maps the features learned by the encoder network to the category label, which is composed of “scale_fc” layer, “FC” layer and “softmax”. The size of the “FC” layer is consistent with the number of output categories to output categories, “softmax” maps the output of multiple neurons to the (0,1) interval, which can be regarded as a probability to understand, so as to carry out multi-classification.

#### 2.3.3. VAE enhancement strategy.

**Fig 9:**
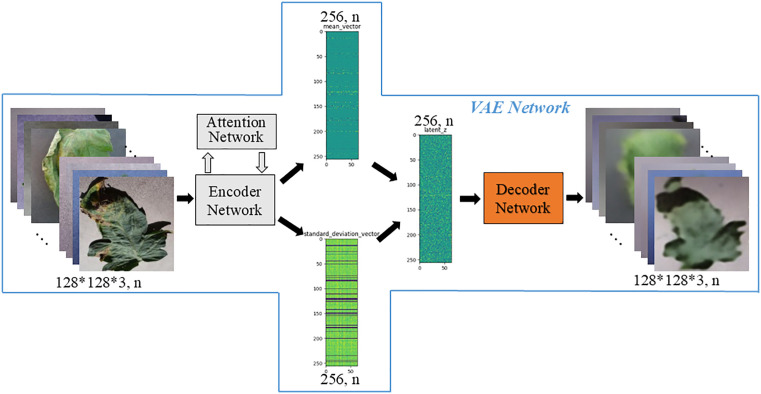
Network model of VAE enhanced.

Compared with other commonly used classification, crop disease identification is a more professional work that requires more experience. For most people, the identification of crop diseases is difficult to accurately judge the types of diseases due to the lack of experience, and seeking experienced experts to identify the types of diseases is a laborious and difficult thing, there is a certain degree of difficulty and can not achieve real-time discrimination. There are two reasons for the error of using convolutional neural network identification, either because the amount of data used to learn is too small to cover the sample space needed to solve the problem, or because the training is too fine, it is impossible to form an effective prediction for new unknown samples or data, that is, overfitting. Aiming at the crop disease recognition in the greenhouse, in the actual research, only a part of the disease pictures are accurately labeled, and most of the disease pictures are not labeled. Therefore, the VAE enhancement strategy is proposed to improve the detection accuracy of the tomato leaf disease identification model based on deep neural network. The parameters of the encoder network are prepared for the following classification, as shown in Fig 9.

It should be noted that our goal is to strengthen the learning of the classification network branch and improve the recognition accuracy. The introduced VAE model branch does not participate in the operation in the test and application phase, but is only used in the training phase, so it will not introduce additional computing and storage consumption in the actual model deployment and production environment. In addition, Gaussian sampling is used in the VAE model and the classification model. In addition to generating coding, noise is also increased, the generalization ability of the model is improved, and overfitting is reduced.

**Fig 10:**
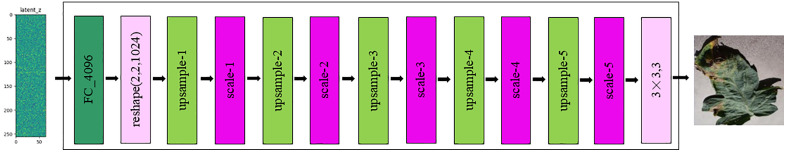
Decoder network.

The VAE Network is divided into three parts: Encoder Network, Attention Network and Decoder Network. The encoder network and the attention network are the encoder network with the attention network shown in Fig 6. The decoder network contains “FC_4096”, “upsample”, “scale”, “conv3 × 3” (representing the standard convolution operation with a convolution kernel of size 3 × 3) structure, the input is a hidden variable of size 256, and the output is a reconstructed image of size 128 × 128 × 3, as shown in Fig 10. The hidden variables are not generated directly from the input data, but are sampled from the Gaussian distribution of the variables calculated from the input data. The Encoder Network is the “intelligent perception core” of the model. Through successive downsampling and convolution, it compresses and abstracts the high-dimensional pixel data of the original image into low-dimensional feature representations rich in semantic information, thereby achieving a deep understanding and efficient representation of the essence of the image content (such as structure, texture, and object categories). This provides a crucial information foundation for subsequent decoding and reconstruction or classification decisions. The core advantage of the Decoder Network lies in its ability to spatially refine and decode the abstract features learned by the Encoder, thereby transforming the perception of “what” into precise predictions of “where” and “how the contour is.” Through upsampling, feature fusion, or attention mechanisms, the Decoder can generate pixel-level semantic segmentation maps, high-precision object bounding boxes, or instance masks, directly enhancing the model's accuracy and fine-grained understanding in object localization, contour delineation, and complex scene comprehension. It is a crucial link in realizing the transition from feature understanding to specific prediction outputs.

“FC-4096” in the decoder network first changes the hidden variable of size 256 into a one-dimensional variable of size 4096 through the fully connected layer, and then transforms the shape to 2 × 2 × 1024, “upsample” uses a 3 × 3 convolution kernel to perform extended convolution operation to realize the expansion of input size and the transformation of the number of channels. “scale” is the structure of Fig 7(a). “conv-3” uses a 3 × 3 convolution kernel to extract features and reduce the channel from 32 to 3. The input is W × H × 32 and the output dimension is W × H × 3, which is the restored image.

#### 2.3.4. Reconstruction-Generation Network(RGN).

##### 2.3.4.1 Model structure:

In order to make the training model adapt to the characteristics of specific data in the agricultural field, a RGN is added based on the attention recognition model. The basic structure of the model is shown in Fig 4(d). The RGN can force the classification network to learn the discriminative regional features in the picture instead of paying more attention to the global features. Since the reconstruction-generation method is only used during training, it does not increase the complexity of the network and does not participate in the operation in the inference stage, so it will not introduce additional computational overhead and storage consumption in the actual model deployment and production environment. The RGN is used to enhance the difficulty of fine-grained recognition and train the classification model to obtain expert knowledge. The identification model consists of three parts. The red box is the identification network, which is used in both training and inference, the blue box and the green box are the discrimination network and the RGN, respectively. These two models are only used in training and not used in inference, so they will not increase the time cost of inference.

**Fig 11:**
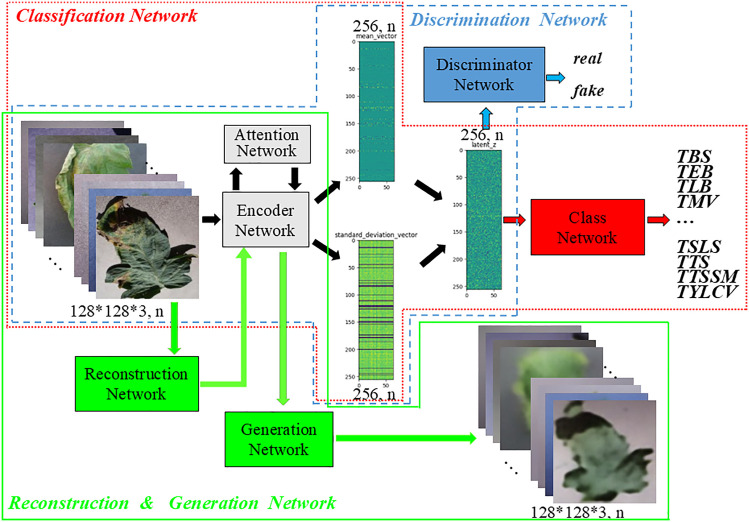
Identification model based on reconstruction-generation.

The structure of the RGN is shown in Fig 11, and the identification network is the structure shown in Fig 4(b). The crop disease recognition is regarded as a fine-grained classification problem, and the attention mechanism is introduced into the disease recognition model. VAE enhancement strategy is first adopted during training to improve the recognition accuracy when the annotation is insufficient, and then through the RGN, The classification network is forced to pay more attention to the discriminative region to find the difference, and the adversarial loss is applied in the discriminative network to distinguish between the generated image and the original image. RGNThe RGN is designed to compel the model to focus on discriminative local details of an image, rather than relying solely on global features. This approach prioritizes fine-grained, class-relevant particulars over holistic information by adopting a “destroy first, rebuild later” methodology. The architecture consists of a Reconstruction Network and a Generation Network. The process begins with a “destruction” phase, where the input image is partitioned into numerous local regions and then reassembled. To mitigate the noise introduced by this disruption and reorganization, a Discrimination Network is employed to distinguish the generated image from the original, thereby suppressing artificial noise patterns. Furthermore, to accurately reconstruct the image, a region alignment network is utilized to restore the original spatial layout of the regions. The Discrimination Network leverages hidden features derived from the recognition network to form a powerful discriminator. This dual objective not only enhances the network's generative capabilities but also significantly improves its feature extraction performance.

RGNThe RGN is built upon the principle of self-supervised learning. Self-supervised learning is a paradigm in which models generate their own supervisory signals from unlabeled data, eliminating the need for manual annotation. The core idea is to create pretext tasks that force the model to learn meaningful data representations by predicting hidden or transformed parts of the input. For instance, in computer vision, common pretext tasks include predicting the rotation angle of an image or reconstructing occluded portions. In natural language processing, a widely used technique is masked language modeling, where the model learns by predicting intentionally masked words or phrases within a sentence, as seen in models like BERT. The primary advantage of self-supervised learning is its ability to leverage vast amounts of unlabeled data. By learning the underlying structures and patterns of the data, the model derives high-quality feature representations that can be effectively transferred to downstream tasks such as classification and regression. Furthermore, since it does not rely on human-provided labels, this approach inherently avoids issues related to incomplete or erroneous annotations.

Since the RGN is only used during training, it does not increase the complexity of the model, and does not require any additional knowledge during recognition. Except for the feedforward stage of the standard classification network, the model does not participate in the operation in the inference stage, which will not introduce additional computational overhead and storage consumption in the actual model deployment and production environment. Compared with the traditional classification network model, the generalization ability of the proposed method is enhanced, and the recognition accuracy is further improved.

##### 2.3.4.2. Each component module:

The RGN is mainly composed of encoder network “E”, attention network “A”, reconstruction network “R” and generation network “G”. The RGN is a fixed operation process without training, which is used to disrupt the spatial distribution of local areas. Firstly, the input image is divided into many local regions, and then they are shuffled and recombined into a single image by the reorganization mechanism.

For an image I of size 128 × 128, divide it into N×N subregions (take N=8), and each region is labeled with $R_{ij}$, and for row i and column j indices, 1<i,j<N respectively, to shuffle these local regions within the flat region. Specifically, generate a random vector $q_{j}$ for the first row j of R, where the value of the i element is $q_{j,i=i+r}$, r is a random variable in the range [−k,k], obeying the uniform distribution of U(k,−k). k is an adjustable parameter 1≤k<N that defines the adjacent range of the transformable region, here set to 2. A new arrangement of the regions in the j row can then be obtained by sorting the array $q_{j}$. This shuffling method destroys the global structure and ensures that the local regions are regrouped within an adjustable range. Since the global structure is broken, in order to recognize these randomly shuffled images, the classification network must seek discriminative regions and then learn the subtle changes in these categories. In practice, the method of first rearranging and then rearranging the rows is used, that is, after dividing the image into N columns, the columns in the same row are swapped, after all the rows are swapped, the rows in the same column are swapped, and then the images are reassembled according to the rearranged array.

**Fig 12:**
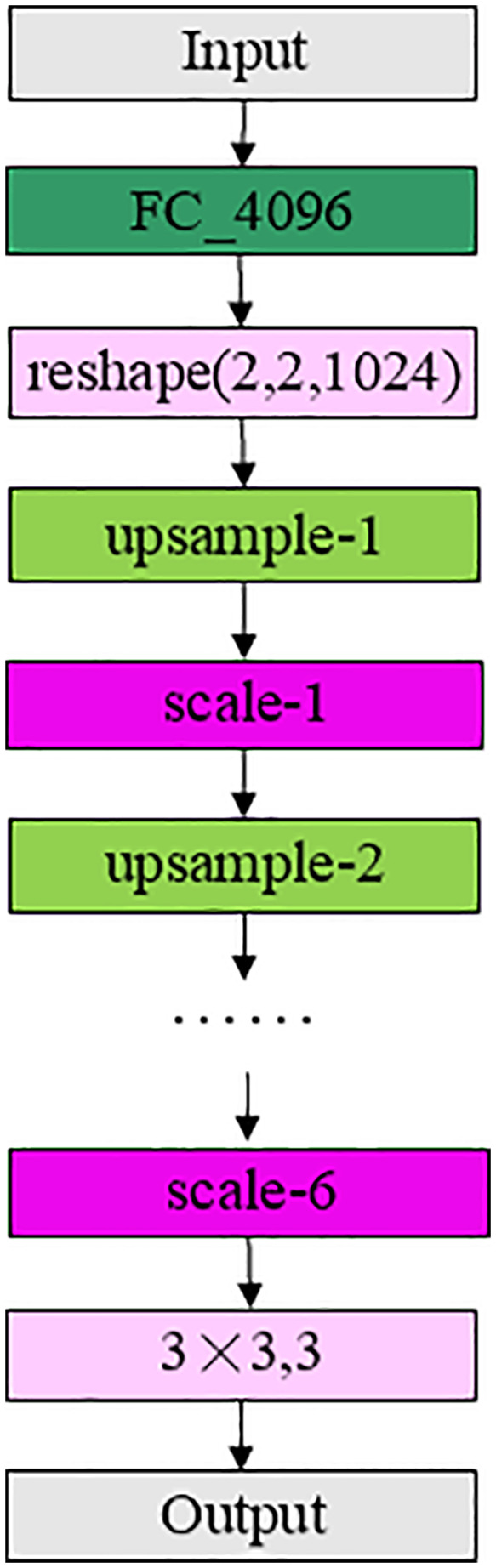
Generation network.

The generation network is as Fig 12. And the discrimination network is mainly composed of three parts: encoder network “E”, attention network “A”, and discrimination network “D”. “D” will be able to distinguish the generated image from the real image as much as possible, so the score of the original image should be as high as possible, and the score of the generated image should be as low as possible. The discriminator network consists of a “scale_fc” followed by a “FC” layer of size 2 to output fake or real images.

## 3. Results and discussion

### 3.1. Experimental setup

Batch training method was used, and the training set and test set were divided into multiple batches. Each batch is trained with 32 images, that is, the minimum batch (minibatch) is set to 32. After training 4096 images, the validation set was used to determine the retention model. After training all the training set images, the test set was tested, and each test batch was set to 32. All the pictures in the training set are iterated as one iteration (epoch) for a total of 10 iterations. The momentum optimization algorithm was used to optimize the model, and the learning rate was set to 0.001.

### 3.2. Training and application of identification model

The process of recognition model training and application includes two stages: training the recognition model and applying the identification model. In training, we first train the VAE network with labeled and unlabeled data, and then use the labeled data to train the reconstruction-generation-based disease identification model. In the application, we only use the trained “classification network” to recognize the labels of the input images. In order to verify the usability of the recognition model in different scenarios, the data set is divided into five groups according to the ratio of training set and validation set, as shown in Table 1, and the sequence numbers 1–5 correspond to the ratio of training set and validation set of five samples, respectively.

### 3.3. Analysis of classification results

#### 3.3.1. Model comparison and analysis.

Recently, ViT(Vision Transformer) [13, 14] successfully applied the transformer architecture to image classification tasks, achieving great success, often outperforming convolutional neural networks. However, the high performance of ViT depends on the use of large datasets, and its dependence on large datasets has been interpreted as due to low local inductive bias [15]. In contrast, ViT performs less well on small-scale datasets than CNNS because ViT lacks the inductive bias, locality, and hierarchical structure representations commonly found in CNNS. ViT therefore requires large-scale dataset pre-training to learn the inductive bias from it, e.g., JFT300M [16]. Existing methods try to lure the necessary inductive bias through structure design and loss function construction, but these methods are not flexible enough and sensitive to the distribution of data. Vits rarely use convolutional filters, which are at the heart of CNNS. This leads to a lack of local inductive bias in the structure of ViTs, requiring large amounts of training data to obtain acceptable visual representations.

To alleviate the burden of pre-training, several ViTs that can learn from scratch medium-size datasets have been proposed in an attempt to increase the locality induction bias in terms of network architecture. These approaches include adopting hierarchical architectures like CNNS to leverage various receptive fields [13] [17], or modifying self-attention mechanisms [18, 19]. However, learning small datasets from scratch is still challenging, as learning for medium-size datasets still requires significant cost considering the trade-off between dataset capacity and performance. Table 3 shows that ViT can achieve good results when the labeled training set has a certain size, but when the data set is small, such as serial number No.1–2, some ViT methods are not satisfactory. Compared with the pure multi-scale recognition network model, the disease recognition accuracy is also lower. The difference is more obvious, indicating that the proposed multi-scale recognition model has a better effect on small-scale data sets. When the VAE enhanced strategy is used, the detection accuracy can be more greatly submitted, which can be extended to similar applications such as crop leaf disease recognition with small amount of data.

**Table 3:** Comparison of identification accuracy of different network models.

Model	No.1	No.2	No.3	No.4	No.5
PVT-Small [14]	72.49	84.49	90.49	94.04	95.05
PVT-Medium [14]	76.40	86.85	92.17	95.56	95.81
CvT-21 [17]	75.45	87.48	93.63	96.04	96.29
Swin-T [13]	75.33	86.97	93.34	95.69	95.93
Swin-S [13]	77.14	89.13	94.37	96.62	97.29
Class Model	78.91	90.32	93.27	95.76	96.24
Proposed	90.57	95.97	96.71	97.70	97.89

ViT is a Transformer-based image classification model with good classification performance, excellent transferability, and good interpretability, among others. However, ViT suffers from scalability issues when dealing with larger images, and its computational complexity and space complexity will increase dramatically. In the case of dealing with complex scenes and multiple objects, vit may not perform well enough compared with some classical object detection models. Its good performance depends on large-scale datasets and high-quality annotations, and its performance is limited for small-scale datasets. Different from the image classification models based on traditional convolutional neural networks, ViT needs to divide the input image into a set of small patches and input them as one-dimensional vectors. This means that specific data preprocessing is required, which adds some additional complexity and overhead.

F1 score is a metric used in statistics to evaluate the performance of binary classification models. Its value ranges from 0 to 1. It takes into account both precision and recall, and is a comprehensive indicator for measuring the performance of classification models. It measures the accuracy of the algorithm by balancing precision and recall. This metric balances the weights of precision and recall and is suitable for scenarios where the importance of both is comparable. The formula is as follows:


$$F\text{1}\;Score=\frac{\text{2}\times Precision\times Recall}{Precision+Recall}$$ (1)

Among them, Recall refers to the proportion of samples that are correctly predicted as positive samples out of the total number of positive samples. It indicates which samples that have been labeled as positive examples we have successfully captured. The formula is as follows:


$$Recall=\frac{TP}{TP+FN}$$ (2)

Precision, which refers to the proportion of samples that are correctly predicted as positive samples among all samples predicted as positive, can be calculated using the following formula:


$$Precision=\frac{TP}{TP+FP}$$ (3)

Here, TP represents the number of true samples; FP represents the number of false positive samples; and FN represents the number of false negative samples.

Only the network model adopted in the training process is changed, and the proposed model is compared with other convolutional neural networks. In the experiment, the classification accuracy of the test set is used as the main evaluation index of the experimental results. The higher the classification accuracy, the better the performance of the model and the stronger the generalization ability. Table 4 lists the comparison of leaf disease recognition indicators for the PlantVillage data set divided by 6:2:2 under different neural network models, in which the comparison experiments are all completed under the same conditions, and the Proposed in the table represents the algorithm proposed in this paper.

**Table 4:** Comparison of leaf disease identification accuracy of different models (unit %).

Model	ResNet-50 [20]	MobileNet-V2 [21]	Bilinear CNN [22]	DCL [23] (ResNet-50)	PMG [24] (ResNet-50)	Proposed
accuracy rate	95.32	96.38	96.89	97.61	97.34	98.03
F1 Score	93.10	93.57	94.62	95.31	95.15	97.95

It can be seen from Table 4 that the recognition accuracy of the fine-grained leaf disease recognition model based on reconstruction-generation is improved on the test sets of multiple categories of two different crops, reflecting the effectiveness of using the attention mechanism and reconstruction-generation method to improve the performance of the network model. The improved convolutional neural network was compared with several more advanced convolutional neural network models such as ResNet-50 [20], MobileNet-V2 [21], Bilinear CNN [22], DCL [23] based on ResNet-50, and PMG [24] based on ResNet-50. These models are used to diagnose and identify the leaf diseases of peach and tomato respectively. Table 4 shows that the proposed method has great advantages over other methods in the identification of different leaf diseases, which is 0.69% higher than that of PMG method and 0.42% higher than that of DCL method. Meanwhile, the F1 score is also the highest among the proposed methods.

**Fig 13:**
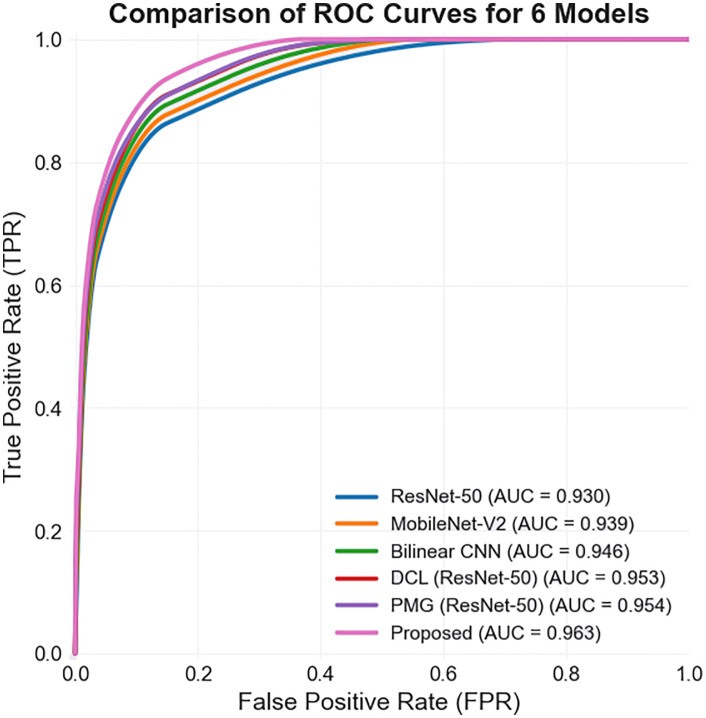
ROC curve.

Receiver Operating Characteristic Curve (ROC Curve) has the horizontal axis representing the false positive rate (FPR) and the vertical axis representing the true positive rate (TPR). For a certain classifier, a pair of TPR and FPR points can be obtained based on its performance on the test samples. Although using the ROC curve to represent the performance of a classifier is intuitive and convenient, people always hope to have a numerical value to indicate the quality of the classifier. Thus, Area Under ROC Curve (AUC) emerged. As the name suggests, the value of AUC is the size of the area under the ROC curve. Usually, the value of AUC ranges from 0.5 to 1.0, and a larger AUC indicates better performance. AUC (Area Under ROC Curve) is a standard used to measure the quality of a classification model (Fig 13).

In the PlantVillage dataset, there are a total of 18,162 tomato leaf images classified into 10 categories. In Fig 1, 18-26 are tomato leaves with bacterial spot(TBS), early blight(TEB), late blight(TLB), leaf mold(TLM), mosaic virus(TMV), septoria leaf spot(TSLS), target spot(TTS), two spotted spider mite(TTSSM) and yellow leaf curl virus diseases(TYLCV) respectively, 38 is tomato healthy leaves(TH). Table 5 presents the confusion matrix for the identification indicators of tomato leaf disease using this method. It clearly shows how many different diseases are classified into other categories. It can be observed from the table that most disease types can be correctly identified, and the number of each disease type being classified into other categories can also be seen.

**Table 5:** Confusion matrix for establishing tomato leaf disease identification indicators using the proposed method.

			*Predicted*								
		TH	TBS	TEB	TLB	TLM	TMV	TSLS	TTS	TTSSM	TYLCV
Real	TH	903	9	15	12	6	3	3	0	3	0
	TBS	18	1215	9	6	9	6	6	3	3	3
	TEB	15	6	567	0	3	0	6	0	0	3
	TLB	12	6	9	1083	12	9	6	3	6	0
	TLM	9	6	3	0	534	9	0	6	3	0
	TMV	6	0	3	0	3	207	0	0	0	3
	TSLS	15	6	3	3	9	6	993	9	12	6
	TTS	3	3	6	9	6	3	3	798	9	3
	TTSSM	12	6	0	6	0	9	3	12	951	6
	TYLCV	21	9	12	15	6	21	24	15	33	3060

#### 3.3.2. Ablation experiment.

The proposed fine-grained leaf disease identification model based on reconstruction-generation adopts the attention mechanism. The model consists of three parts: identification network, discrimination network and RGN. In order to verify the effectiveness of each network, the following ablation experiments are performed, as shown in Table 6, where “i” stands for " identification network” without “attention network,” “a” stands for “attention network,” “V” stands for “VAE network,” “d” stands for “discrimination network,” and “r” stands for “RGN.” “i + a” is the " identification network” including “attention network,” “i + a + V” is the " identification network” enhanced by VAE containing the “attention network,” “i + a + d + r” is the " identification network” including the “discrimination network” and “RGN” containing “attention network,” “i + a + V + d + r” is the “recognition network” with “attention network” enhanced by VAE and “discrimination network” and “RGN,” that is, the fine-grained identification model of greenhouse crop leaf diseases based on reconstruction-generation proposed in this paper. As can be seen from the table, the proposed method has a great improvement.

**Table 6:** Comparison of leaf disease identification accuracy with different modules (unit %).

Model	i	i + a	i + a + V	i + a + d + r	i + a + V + d + r(Ours)
accuracy rate	96.35	96.92	97.24	97.75	98.03
F1 Score	95.55	95.94	96.43	97.21	97.95

**Fig 14:**
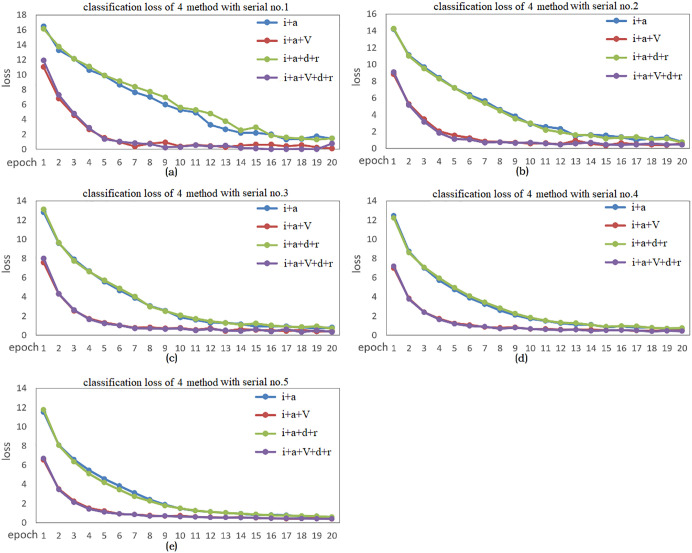
Comparison of indicators for 5 sets of data using different methods. **(a)**-(e) Classification losses corresponding to 5 different samples.

In Fig 14, (a)-(e) correspond to the classification losses of the four methods in 5 different samples. As can be seen from figure, the losses of i + a and i + a + d + r are larger than those of the methods using the enhancement method, and the loss decreases more slowly. This indicates that using the enhancement method results in a smaller loss and a faster decrease. Through comparison, it is known that when the number of labeled samples is small (serial no. 1), the increase in model uncertainty leads to a decrease in accuracy. However, when the number of labeled samples is large (serial no. 3–5), the increase in model uncertainty leads to a slight increase in accuracy. The enhancement strategy can significantly increase the classification accuracy. This is because although Gaussian sampling increases uncertainty, using VAE enhancement enables the model to learn this uncertainty and enhances the model's generalization ability.

### 3.4. Analysis of test results

#### 3.4.1. Detection model.

There are two popular solutions for object detection tasks, one is two-stage object detection, and the other is one-stage object detection. For two-stage object detection, a neural network is first required to identify the object, and then classify the identified object. The benefit of this method is that it is easy to implement, but the downstream classification task depends on the performance of the upstream recognition and positioning task. The one-stage object detection method directly uses the network to detect the object, although it does not need to identify the object first, it increases the difficulty of end-to-end object detection. In general, the two-stage object detection method has high accuracy, but it is not fast. The single-stage detector is fast, but the accuracy is not the highest. In the application scenario of crop disease detection, the detection speed of the two-stage method can meet the requirements and has a high accuracy, such as Faster R-CNN [25]. Therefore, the two-stage object detection method can be used in the study.

Mask R-CNN [26] is proposed by Facebook AI research team in 2017, which adds an object mask branch to Faster R-CNN in parallel with the existing branch to enhance the performance of Faster R-CNN on border recognition. Mask R-CNN is used for object instance segmentation, which not only gives the bounding box containing the object, but also gives the exact segmentation map of the object. Instance segmentation algorithms usually require assigning an accurate pixel-level segmentation mask supervision label to all training samples. However, these labels are very difficult to collect, and labeling new categories is a time-consuming and laborious task. In the Mask R-CNN framework, the input image is first extracted through the backbone network to obtain feature maps of different scales, and then a number of anchor boxes are generated by the RPN network, and multiple regions of Interest (ROI) are retained after non-maximum suppression (NMS). Due to the different stride, the ROI Align operation is performed on the corresponding stride of the feature maps of different scales. The rois generated by this operation are concatenation connected and then divided into three parts: Fully convolutional prediction pixel segmentation (mask), fully connected prediction bounding box (bounding box), and fully connected prediction class (class). That is, its output consists of three parts: the object class, the bounding box surrounding the object, and the pixel segmentation map.

In order to improve the detection accuracy of tomato leaf disease recognition model based on deep neural network, a VAE enhancement strategy is proposed, which is implemented in two steps. Firstly, the multi-scale tomato leaf disease recognition network model based on VAE is trained to obtain the parameters of the encoder model. After that, the trained encoder network was used as the backbone network of Mask R-CNN, the encoder network was used as the backbone network to extract the characteristics of disease leaves, and the parameters were the preliminary trained encoder network parameters, and then the segmentation data of TJ-Tomato disease leaves were used to continue training the model. The model parameters were fine-tuned to achieve accurate detection and segmentation. In actual use, only the Mask R-CNN network is involved in the operation, and the VAE network is only involved in the test phase. Therefore, no additional calculation and storage consumption will be introduced in the actual model deployment and production environment.

#### 3.4.2. Detection results.

The Mask R-CNN framework was used to detect 167 diseased leaf images and 139 healthy leaf images in the test set. A total of 826 tomato leaves were detected in the test, including 243 diseased leaves and 583 healthy leaves. Among the 243 diseased leaves, 12 were incorrectly identified, with a false recognition rate of 4.93%. Among them, only 1 was identified as a healthy leaf, and the other 11 were identified as other diseases, accounting for 0.41% and 4.53% of the total number of diseased leaves detected, respectively, which meant 0.41% of the diseased leaves were mistakenly identified as healthy leaves. 4.53% of the diseased leaves were mistakenly identified as other types of diseases. Nine out of 583 healthy leaves were identified as diseased leaves, with a false recognition rate of 1.54%. In conclusion, the correct recognition rate of diseased leaves was 95.07%, and only 0.41% of diseased leaves were mistakenly identified as healthy leaves. The correct recognition rate of healthy leaves was 98.46%, and only 1.54% of healthy leaves were mistakenly identified as diseased leaves.

The experimental results show that the proposed method has a great improvement and can improve the accuracy for the subtle differences among diseases. The proposed method is applied to the detection model, which can realize the end-to-end detection and identification of diseased leaves and meet the practical requirements.

### 3.5. Discussion

Leaf disease identification is crucial for controlling the spread of diseases and ensuring the healthy development of the tomato industry. Methods based on deep neural networks require a large number of sample images as the training set to improve the generalization ability of the trained model. However, many agricultural fields are data-poor areas. In the field of leaf disease identification, collecting a large number of leaf disease images and accurately annotating the images is a very time-consuming and labor-intensive task. Additionally, precise annotation of data requires professional domain knowledge, which ordinary people cannot accomplish. Due to these factors, the number and categories of annotations are relatively small. Moreover, since manual annotation has a certain degree of subjectivity, it is difficult to have a unified standard to judge the accuracy and completeness of the annotations.

In future work, we will continue to search for better data augmentation methods to solve the problem of tomato leaf disease identification, thereby improving the robustness and accuracy of the identification, and it can be applied to the detection network.

(1) Based on the features of plant leaf disease images, in the early stage when the lesion is not obvious, the disease area is small and the same disease has significant differences at different stages, and the image of the disease-free part is similar to the healthy leaf, the fine-grained image classification method is adopted to improve the recognition accuracy.
(2) Facing the imbalance of the dataset, new collected disease images are used to expand the dataset. For cases where there is a lack of expert annotations, unsupervised learning can be used to fully utilize the unannotated data, and then supervised disease identification can be carried out using the annotated data.
(3) In practical work, due to the difficulty in collecting disease leaf images, the problem of small sample learning urgently needs to be solved.

## 4. Conclusions

Deep neural networks need strong data support, and a large number of agricultural fields are data vacuum areas, which makes the accurate identification of diseases difficult. At the same time, due to the lack of accurate annotation of agricultural disease data, the related work is more difficult. In research, it is often encountered that the dataset has a certain scale, but the amount of annotation is relatively small. How to apply these unlabeled disease data is a problem worth studying. To this end, this paper proposes a fine-grained recognition model for greenhouse crop leaf diseases based on reconstruction-generation.RGN Experimental results show that the classification recognition accuracy of the proposed fine-grained leaf disease identification model based on reconstruction-generation adopts the attention mechanism reached 98.03%. The proposed method is applied to the detection model, the correct recognition rate of diseased leaves was 95.07%, and the correct recognition rate of healthy leaves was 98.46%, which can realize the end-to-end detection and identification of diseased leaves and meet the practical requirements.

This paper conducts research on the crop disease recognition technology in greenhouse scenarios, and the proposed method has enhanced generalization ability and further improved recognition accuracy. In addition, while the accuracy of the model is improved compared to other methods, the number of parameters is reduced. Integrating it into mobile applications for use on mobile devices is feasible, such as integrating it into smart phones, drones, and other automatic agricultural vehicles for growers or agronomists to use, to achieve real-time monitoring and disease detection of large-scale cultivated crops. The model can identify plant leaf diseases, meeting the needs of disease identification in agricultural production, providing a theoretical basis for the subsequent development of intelligent plant leaf disease recognition devices, and can be extended to other similar application scenarios for crop disease recognition.

With the continuous improvement of artificial intelligence technology and sensor technology, as well as the continuous development of image analysis processing technologies and algorithms, the monitoring methods for crop diseases based on image processing will continuously move towards practical applications. Future research will mainly focus on the following aspects: 1) Conducting early disease detection research. Since some symptoms of crops may be associated with the symptoms of diseases in the early stage, but they may not necessarily develop into diseases in the future, precise disease annotation cannot be achieved at this time, and the training samples are relatively few. Therefore, the weak supervision idea can be applied to the early crop disease recognition. Research on early prediction and diagnosis models, establishing an early warning mechanism, and early diagnosis and prevention will likely promote the progress of early crop diagnosis in practical applications, and will be more significant than the identification of diseases in the middle and later stages. 2) Facing the imbalance of data sets, expanding the data set with newly collected disease images. For cases where there is a lack of expert annotations, unsupervised learning can be used to fully utilize the unannotated data, and then supervised disease recognition can be carried out using the annotated data. In practical work, due to the difficulty in collecting disease leaf images, the problem of small sample learning urgently needs to be solved. 3) Since the proposed method meets the accuracy requirements of crop leaf disease image recognition while requiring lower computing power, it can run on low-performance terminals. Therefore, in the next step, it can be integrated into mobile applications for use on mobile devices, such as integrating it into smart phones, drones, and other automatic agricultural vehicles for growers or agronomists to use, to achieve real-time monitoring and disease detection of large-scale cultivation.
